# Nomophobia among university students: Prevalence, correlates, and the mediating role of smartphone use between Facebook addiction and nomophobia

**DOI:** 10.1016/j.heliyon.2023.e14284

**Published:** 2023-03-07

**Authors:** Firoj Al-Mamun, Mohammed A. Mamun, Md. Salauzzaman Prodhan, Md. Muktarul, Mark D. Griffiths, Mohammad Muhit, Md. Tajuddin Sikder

**Affiliations:** a CHINTA Research Bangladesh, Savar, Dhaka, 1342, Bangladesh; b Department of Public Health and Informatics, Jahangirnagar University, Savar, Dhaka, 1342, Bangladesh; c Department of Public Health, University of South Asia, Dhaka, Bangladesh; d Department of Statistics, Jahangirnagar University, Savar, Dhaka, 1342, Bangladesh; e Psychology Department, Nottingham Trent University, 50 Shakespeare Street, Nottingham, NG1 4FQ, United Kingdom

**Keywords:** Nomophobia, Smartphone addiction, Facebook addiction, Depression

## Abstract

Nomophobia (‘no mobile phone phobia’) has been growing issue worldwide in recent years and has been associated with a number of psychological and behavioral health-related problems. However, few studies have examined nomophobia in Bangladesh. Therefore, the severity and correlates of nomophobia, and the mediating role of smartphone use between Facebook addiction and nomophobia was investigated. A cross-sectional study utilizing 585 university students was conducted employing a convenience sampling method. Data were collected using a survey in March 2022. The survey comprised questions related to socio-demographics, behavioral health, academic performance, nomophobia, smartphone addiction, Facebook addiction, insomnia, and depression. The mean score of nomophobia was 88.55 out of 140 (±21.71). The prevalence was 9.4% for mild nomophobia, 56.1% for moderate nomophobia, and 34.5% for severe nomophobia. First-year students had higher levels of nomophobia than other years. Significant predictors for nomophobia included daily duration of smartphone time, psychoactive substance use, and being in a relationship. Nomophobia was significantly associated with smartphone addiction, Facebook addiction, insomnia, and depression. Moreover, smartphone addiction significantly mediated the relationship between Facebook addiction and nomophobia. Strategies that help reduce daily smartphone time, and reduce psychoactive substance use might help reduce nomophobia prevalence among university students.

## 1 Introduction

Digital technologies have significantly changed the lives of individuals by facilitating network access, communication, collaboration, and online education. For many, smartphones have become a necessity in life and their impact on daily lives has been substantial especially among young people [1]. However, researchers have emphasized that smartphones could lead to potentially dangerous antisocial and addictive behavior [2]. Excessive and problematic cell phone use leads to nomophobia (i.e., the fear of being without a mobile phone) which some have claimed is a new type of psychiatric disorder [3]. Nomophobia appears to be prevalent in contemporary society. For instance, a systematic review reported prevalence rates for nomophobia being between 13% and 79% [4], and a meta-analysis reported the pooled prevalence of moderate to severe nomophobia to be 70.76% [5].

Nomophobia has been increasingly studied among university students globally. For example, a study among university students of Oman (n = 735) reported that all of the students had nomophobia More specifically, the prevalence was 20% for mild nomophobia (scoring 21–59 out of 140 on the Nomophobia Questionnaire [NMP-Q]), 15% for moderate nomophobia (scoring 60–99 out of 140 on the NMP-Q), and 65% for the severe nomophobia (scoring 100–140 out of 140 on the NMP-Q) [6]. Similarly, a study of Indian university students (n = 209) reported 8.1% mild nomophobia, 56.5% moderate nomophobia, and 35.4% severe nomophobia [7], whereas among Saudi Arabian university students (n = 625), the rate was 63.2% mild nomophobia, and 22.1% severe nomophobia, both studies using the NMP-Q [8]. Another study conducted among Indian physiotherapy students (n = 157) reported that the mean score of nomophobia using the NMP-Q was 77.6 [9].

University students have been used as samples to investigate nomophobia in different countries such as India [7], Saudi Arabia [8], Oman [6], Pakistan [10], Ghana [11], and Kuwait [12]. In general, they have reported a relatively elevated level of severe nomophobia among university students ranging from 22.1% to 65%. Previously, it was reported that nomophobia is associated with various smartphone-related factors such as duration of using it [8,13], checking smartphone frequently, daily incoming and outgoing calls and messages [13], checking a smartphone without a purpose, and using a smartphone immediately on waking up [14]. Additionally, it has been reported that nomophobia has significant associations with anxiety [15], depression [16], low self-esteem [17], hyperactivity and oppositional problems [18], and stress [19]. However, to date, only one small-scale study has been carried out in Bangladesh (n = 132 undergraduates). The study reported that 7.6% had mild nomophobia, whereas 68.9% had moderate nomophobia, and 18.9% had severe nomophobia using the NMP-Q [20]. The study did not report any association between nomophobia and smartphone addiction, Facebook addiction, depression, and insomnia, and did not use any model to identify the predictive factors. Therefore, the present study examined the prevalence and correlates of nomophobia using a much larger Bangladeshi university student sample.

Previous studies have reported a relationship between nomophobia, smartphone addiction (SA), and Facebook addiction (FA). For example, a significant positive association has been reported between nomophobia and SA among Philippines high school students [21], Turkish adolescents [22], and Turkish nursing students [23]. Increased addiction to Facebook among adolescent students in Morocco was reported in parallel with nomophobia scores [24]. Additionally, research had suggested that FA and SA share some common risk factors such as impulsiveness, and lack of social support [25]. Furthermore, it has been asserted that nomophobia and SA share symptoms such as long-term smartphone use, frequently checking texts and calls, carrying a smartphone in every place, never switching off the smartphone, feeling anxious regarding the potential loss of a smartphone, and communicating virtually rather than face-to-face interactions [[26], [27], [28]], and they are mutually affected by each other [28]. However, it is unclear whether SA mediates the relationship between FA and nomophobia. Therefore, based on the aforementioned literature, the present study hypothesized that (i) FA would have a direct effect on nomophobia and SA, and that (ii) SA would mediate the relationship between nomophobia and FA.

## 2 Methods

### 2.1 Study procedure and data collection

A cross-sectional survey study was conducted in March 2022 among the university students of Bangladesh utilizing convenience sampling. Data were collected offline on campus (using a ‘paper and pencil’ survey) and participants were recruited by visiting every faculty or institution, and residence hall. Data from 585 participants were collected after excluding incomplete surveys. Given that around 640 participants were approached, the response rate was approximately 91%. Inclusion criteria were being an undergraduate or postgraduate student of the university and being a smartphone user. Participants were excluded if they did not meet the inclusion criteria. The participants took approximately 30 min to complete the survey comprising 70 items in total.

### 2.2 Measures

#### 2.2.1 Socio-demographic information

Participants’ age, gender, relationship status, monthly family income, the field of study, and information related to the academic year was included in socio-demographic characteristics. Details are reported in Table 1.

**Table 1:** Distribution of the study variables and nomophobia.

Study variables	Total (n, %)	Mean ± SD (NMP-Q) 88.55 ± 21.71	F/*t* (*p*-value)
**Socio-demographic information**			
Age (Mean ± SD)		22.52 ± 1.53	
Gender			
Male	265; 45.3%	88.96 ± 21.59	0.414 (0.679)
Female	320; 54.7%	88.21 ± 21.83	
**Relationship status**			
Single	431; 73.7%	87.69 ± 21.81	−1.601 (0.110)
In a relationship	154; 26.3%	90.95 ± 21.31	
**Monthly family income (BDT)**			
Less than 15000	78; 13.3%	85 ± 22.42	2.006 (0.135)
15000 to 30000	494; 84.4%	89.29 ± 21.53	
More than 30000	13; 2.2%	81.61 ± 22.38	
**Faculty/Institute**			
Arts and Humanities	125; 21.4%	89.19 ± 21.84	1.143 (0.330)
Mathematical Sciences	137; 23.4%	88.34 ± 20.48	
Social Sciences	146; 25%	87.88 ± 22.71	
Biological Sciences	97; 16.6%	91.96 ± 21.58	
Business Studies	32; 5.5%	89.12 ± 17.49	
Law	13; 2.2%	77.92 ± 18.49	
IIT	3; 0.5%	103 ± 7.81	
Remote sensing	2; 0.3%	79 ± 1.41	
BICLC	24; 4.1%	84.45 ± 29.61	
IBA	5; 0.9%	75.20 ± 7.12	
**Academic year**			
First-year	22; 3.8%	94.59 ± 22.31	3.497 (0.008)
Second-year	269; 46%	91.62 ± 20.33	
Third-year	124; 21.2%	85.18 ± 22.92	
Fourth-year	148; 25.3%	85.32 ± 22.47	
Master's	22; 3.8%	85.63 ± 20.20	
**Behavior-related information**			
*Smoked cigarettes*			
No	506; 86.5%	89.20 ± 21.22	1.830 (0.068)
Yes	79; 13.5%	84.40 ± 24.31	
*Drank alcohol*			
No	546; 93.3%	89.04 ± 21.61	0.309 (0.758)
Yes	21; 3.6%	87.57 ± 17.26	
*Used psychoactive substances (ganja, heroin, yaba)*			
No	552; 94.4%	88.92 ± 21.52	2.751 (0.065)
Yes	22; 3.8%	82.54 ± 23.54	
Sometimes	9; 1.5%	74.77 ± 22.76	
*Went to the toilet with a smartphone*			
No	463; 79.1%	87.88 ± 21.70	1.951 (0.143)
Yes	104; 17.8%	90 ± 21.73	
Sometimes	18; 3.1%	97.38 ± 19.67	
*Daily number of hours spent on smartphone* (Mean ± SD)		5.50 ± 2.91	
**Academic-related information**			
*Received lower grades on the class test or important project*			
No	232; 39.7%	86.81 ± 22.07	−1.575 (0.116)
Yes	349; 59.7%	89.69 ± 21.26	
*Received lower grades in an academic course*			
No	239; 40.9%	86.83 ± 22.38	−1.755 (0.080)
Yes	341; 58.3%	90.05 ± 21.04	
*Given course improvement exam or dropped year*			
No	517; 88.4%	89.23 ± 21.42	1.956 (0.051)
Yes	62; 10.6%	83.51 ± 24.27	
*Past year CGPA* (Mean ± SD)		3.45 ± 0.47	

Note: IIT=Institute of Information Technology; BICLC=Bangabandhu Institute of Comparative Literature and Culture; IBA=Institute of Business Administration; CGPA=Cumulative Grade Point Average.

#### 2.2.2 Behavior-related information

Information related to cigarette smoking (yes/no), alcohol drinking (yes/no), psychoactive substance use (i.e., heroin, ganga, yaba; yes/no), and daily smartphone time (in hours) was asked for. Additionally, they were asked if they took a mobile phone into the toilet with them (answered yes, no or sometimes).

#### 2.2.3 Academic-related information

Information was asked for relating to whether they had received a low-grade in-class test/course exam, took an improvement exam and/or had dropped a year, and their past year's CGPA (cumulative grade point average).

#### 2.2.4 Nomophobia

The 20-item Nomophobia Questionnaire (NMP-Q) was used to assess nomophobia [29]. The scale comprises four dimensions comprising (i) *not being able to communicate;* (ii) *losing connectedness,* (iii) *not being able to access information,* and (iv) *giving up convenience,* Items (e.g., “*I would feel nervous because I could not instantly communicate with my family and/or friends”*) are rated on a seven-point scale from 1 (*strongly disagree*) to 7 (*strongly agree*). Total scores range from 20 to 140 and classified as: 0–20 = no nomophobia; 21–59 = mild nomophobia; 60–99 = moderate; nomophobia; and 100–140 = severe nomophobia. The scale showed excellent reliability (α = 0.945).

#### 2.2.5 Smartphone addiction

The six-item Smartphone Application-Based Addiction Scale (SABAS) was used to assess smartphone addiction [30]. The scale comprises six components (i.e., salience, mood modification, tolerance, withdrawal symptoms, conflict, and relapse) [31] and items (e.g., “*My smartphone is the most important thing in my life”)* are rated on a six-point scale from 1 (*strongly disagree*) to 6 (*strongly agree*). Total scores range from 6 to 36 where higher scores indicate greater risk of addiction to smartphone applications. The scale showed acceptable reliability (α = 0.78).

#### 2.2.6 Facebook addiction

The six-item Bergen Facebook Addiction Scale (BFAS) was used to assess Facebook addiction [32]. The scale comprises six components (i.e., salience, mood modification, tolerance, withdrawal symptoms, conflict, and relapse) [31] and items (e.g., *“How often in the last year have you spent a lot of time thinking about Facebook or planned use of Facebook?”*) are rated on a five-point scale (1 = very rarely to 5 = very often). Total scores range from 6 to 30 where higher scores indicate greater risk of Facebook addiction. The scale showed good reliability (α = 0.86).

#### 2.2.7 Insomnia

The seven-item Insomnia Severity Index (ISI) was used to assess insomnia [33,34]. Items (e.g., *“Difficulty falling asleep”*) are rated on a five-point scale from 0 (*not at all*) to 4 (*very severe*). Total scores range from 0 to 28 with higher scores indicating greater insomnia. The scale showed good reliability (α = 0.86).

#### 2.2.8 Depression

The nine item Patient Health Questionnaire-9 (PHQ-9) was used to assess depression [35,36]. Items (e.g., *“Little interest or pleasure in doing things?”*) are rated on a four-point scale from 0 (*not at all*) to 3 (*more than half of the days*). Total scores range from 0 to 27 where higher scores indicate greater depression. The scale showed good reliability (α = 0.86).

### 2.3 Ethics

Informed consent was provided by the participants before data collection. They were notified about the study's nature and purpose with a right to withdraw from the study at any time. Participants were assured about their data confidentiality. No incentives were provided to participate in the study. The study was conducted adhering to the Helsinki Declaration guidelines and the study was approved by the department of Public Health & Informatics, Jahangirnagar University.

### 2.4 Statistical analysis

The collected data were first entered manually into *Google Forms,* and an *Excel* spreadsheet was created to export the responses for data cleaning, and preparing the data for analysis. Data were formally analyzed using the SPSS (Statistical Package for Social Science) version 25, and SPSS AMOS (Analysis of Moment Structure) version 23. Descriptive statistics were computed using SPSS. The data were normally distributed. Independent *t*-tests and one-way analyses of variance (ANOVAs) were performed to investigate the differences between the study variables and nomophobia score. A multiple hierarchical linear regression was conducted utilizing nomophobia as the dependent variable. Furthermore, structural equation modelling-based mediation analysis was performed to examine the hypothesized relationship using AMOS v.23. The total effect, direct effect, and indirect effect were calculated with 5000 bootstrapping samples and a 95% bias-corrected confidence interval. All the test statistics used a *p*-value <0.05 for statistical significance.

## 3 Results

### 3.1 Participant characteristics

**Fig. 1:**
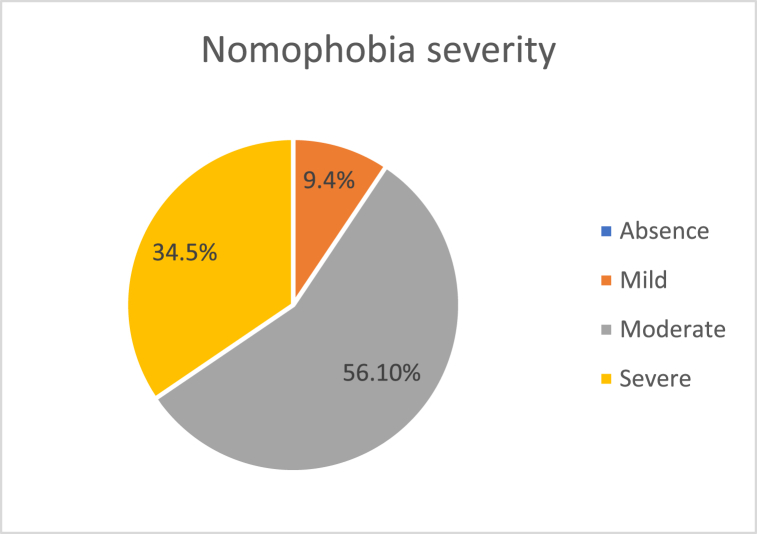
Severity of nomophobia.

The demographic characteristics of the participants are reported in Table 1. The overall prevalence of nomophobia is shown in Fig. 1. It was found that 9.4% of participants had mild nomophobia, 56.1% had moderate nomophobia, and 34.5% had severe nomophobia (Fig. 1).

### 3.2 Mean difference between the study variables and the nomophobia score

Results showed participants’ academic year and nomophobia scores significantly differed. First-year students had the highest nomophobia score compared to students in other academic years (F = 3.497, *p* = 0.008). No significant difference was reported in terms of behavior-related, and academic information and nomophobia score.

#### 3.2.1 Correlation between nomophobia and study scales

Table 2 presents the correlation coefficients between the NMP-Q and the study's other psychometric scales (Table 2). Results indicated that nomophobia had significantly moderate correlations with SA (r = 0.592, *p* < 0.001), and FA (r = 0.420, *p* < 0.001), and a significant (but weaker) relationship with insomnia (r = 0.267, *p* < 0.001), and depression (r = 0.259, *p* < 0.001).

**Table 2:** Pearson correlation coefficients among the study's psychometric scales.

Variables	Mean ± SD	1	2	3	4	5	6	7	8	9
NMPQ Factor 1 (1)	24.27 ± 6.77	1								
NMPQ Factor 2 (2)	24.81 ± 8.00	0.584^a^	1							
NMPQ Factor 3 (3)	24.62 ± 6.76	0.598^a^	0.625^a^	1						
NMPQ Factor 4 (4)	14.83 ± 4.17	0.516^a^	0.354^a^	0.464^a^	1					
NMPQ total (5)	88.55 ± 21.71	0.838^a^	0.840^a^	0.860^a^	0.648^a^	1				
SABAS (Smartphone addiction) (6)	20.79 ± 5.71	0.453^a^	0.553^a^	0.510^a^	0.346^a^	0.592^a^	1			
BFAS (Facebook addiction) (7)	15.12 ± 5.83	0.256^a^	0.443^a^	0.400^a^	0.186^a^	0.420^a^	0.454^a^	1		
ISI (Insomnia) (8)	10.76 ± 6.20	0.153^a^	0.241^a^	0.277^a^	0.167^a^	0.267^a^	0.359^a^	0.493^a^	1	
PHQ (Depression) (9)	9.38 ± 5.61	0.156^a^	0.267***	0.269^a^	0.088^a^	0.259^a^	0.358^a^	0.493^a^	0.571^a^	1

a Correlation is significant at the 0.01 level; * Correlation is significant at the 0.05 level; SABAS = Smartphone Application-Based Addiction Scale; BFAS = Bergen Facebook Addiction Scale; ISI = Insomnia Severity Index; PHQ = Patient Health Questionnaire.

### 3.3 Predictive factors of nomophobia

Predictive models for nomophobia score were tested. Model 1 included socio-demographic information and explained 3% of the variance. Model 2 included socio-demographic information and behavior-related information, and explained 8.1% of the variance. Model 3 included socio-demographic information, behavior-related information, and academic information-related information, and explained 10.1% of the variance. The final model also reported significant predictive factors for nomophobia. These were being in a relationship (coefficient = 5.479, *p* = 0.035), psychoactive substance use (coefficient = 10.048, *p* = 0.014), and greater daily number of hours spent on smartphone (coefficient = 1.427, *p* = 0.001) (Table 3).

**Table 3:** Predictive models for nomophobia.

Variables	Model 1				Model 2				Model 3			
	[R^2^ = .035, adjusted R^2^ = .019, F = 2.119, *p* = 0.051]				[R^2^ = .081, adjusted R^2^ = .052, F = 2.755, *p* = 0.002]				[R^2^ = .101, adjusted R^2^ = .061, F = 2.531, p = 0.001]			
	B	S.E.	*t*	*p*	B	S.E.	*t*	*p*	B	S.E.	*t*	*p*
Constant	90.43	24.08	3.754	.000	92.00	23.97	3.837	.000	105.83	26.16	4.045	.000
Age	−.48	1.15	−.421	.674	−.68	1.14	−.596	.552	−.66	1.16	−.574	.567
Gender^a^	.86	2.40	.360	.719	−.05	2.63	−.019	.984	.35	2.68	.133	.895
Relationship Status^b^	7.25	2.58	2.808	.005	5.80	2.58	2.243	.026	5.47	2.58	2.122	**.035**
Monthly family income^c^	.61	3.05	.201	.841	−.82	3.09	−.267	.790	−1.43	3.11	−.460	.646
Faculty/Institute^d^	−1.17	.56	−2.072	.039	−.98	.56	−1.739	.083	−.83	.56	−1.480	.140
Academic year^e^	.06	1.76	.037	.971	.26	1.74	.154	.878	.64	1.77	.361	.719
Smoking cigarettes^f^					.06	3.87	.016	.988	.31	3.88	.080	.936
Drinking alcohol^f^					1.60	6.19	.259	.796	1.12	6.20	.181	.856
Substance use^g^					−9.97	4.08	−2.439	.015	−10.04	4.08	−2.458	**.014**
Going to the toilet with smartphone^g^					2.04	2.48	.826	.409	2.10	2.50	.837	.403
Daily smartphone use (hours)					1.45	.41	3.521	.000	1.42	.41	3.438	**.001**
Received low grades for in-class tests or project^f^									.79	2.81	.284	.777
Received low grades in academic courses^f^									4.436	2.86	1.551	.122
Given course improvement exam and/or dropped a year^f^									−5.498	3.86	−1.421	.156
Past-year CGPA									−5.036	3.90	−1.289	.198

a 1 = Male, 2 = Female.

b 1 = Single, 2 = In a relationship.

c 1 = Less than 15000 BDT, 2 = 15000 to 30000 BDT, 3 = More than 30000 BDT.

d 1 = Arts and Humanities, 2 = Mathematical Sciences, 3 = Social Sciences, 4 = Biological Sciences, 5 = Business Studies, 6 = Law, 7 = Institute of Information Technology, 8 = Remote sensing, 9 = BICLC (Bangabandhu Institute of Comparative Literature and Culture), 10 = Institute of Business Administration.

e 1 = First year, 2 = Second year, 3 = Third year, 4 = Fourth year, 5 = Master's.

f 0 = No, 1 = Yes.

g 0 = No, 1 = Yes, 2 = Sometimes; CGPA = cumulative grade point average.

### 3.4 Mediation analysis

**Fig. 2:**
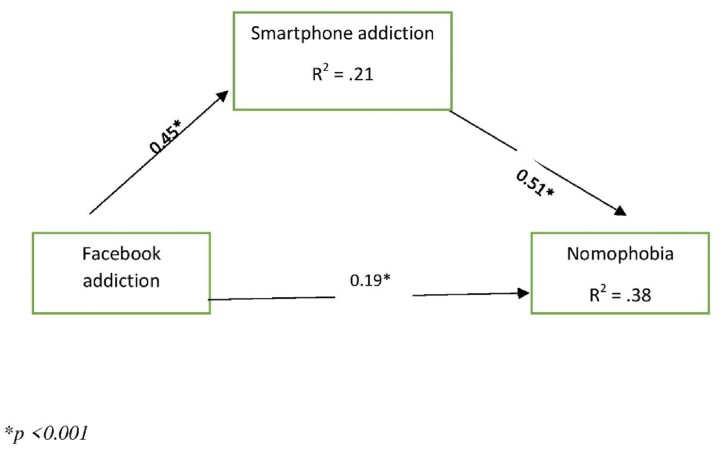
Structural equation modeling (SEM).

Table 4 presents the SEM-based mediation analysis showing that all of the effects were significant. More specifically, FA had a direct effect on SA (β = 0.454, 95% CI: 0.374–0.525, *p* < 0.001) and nomophobia (β = 0.191, 95% CI: 0.109–0.272, *p* < 0.001). SA had a significant direct effect on nomophobia (β = 0.506, 95% CI: 0.421–0.580, *p* = 0.01). Additionally, SA significantly mediated the relationship between FA and nomophobia (β = 0.230, 95% CI: 0.171–0.287, *p* = 0.001). Moreover, FA and SA together explained 38% of the variance in nomophobia, whereas 21% of the variance in SA was explained by FA (Fig. 2).

**Table 4:** Structural equation modeling of nomophobia, Facebook addiction, and smartphone addiction.

Direct, indirect, and total effects	β	SE	LLCI	ULCI	*p*
**Direct effects**					
Facebook addiction → Smartphone addiction	0.454	0.038	0.374	0.525	<0.001
Smartphone addiction → Nomophobia	0.506	0.040	0.421	0.580	0.001
Facebook addiction → Nomophobia	0.191	0.042	0.109	0.272	<0.001
**Indirect effects**					
Facebook addiction →Smartphone addiction→ Nomophobia	0.230	0.030	0.171	0.287	0.001
**Total effects**					
Facebook addiction → Smartphone addiction	0.454	0.038	0.374	0.525	<0.001
Smartphone addiction → Nomophobia	0.506	0.040	0.421	0.580	0.001
Facebook addiction → Nomophobia	0.420	0.034	0.350	0.480	<0.001

β = standardized regression weights; SE = standard error; LLCI = lower level of confidence interval; ULCI = upper level of confidence interval.

## 4 Discussion

The present study investigated the prevalence and correlates of nomophobia, and the mediating role of smartphone addiction between Facebook addiction and nomophobia among a university student sample in Bangladesh. The present study found that 9.4% of participants had mild nomophobia, 56.1% had moderate nomophobia, and 34.5% had severe nomophobia. The study's findings can only be compared with one previous study in Bangladesh which reported that 7.6% had mild nomophobia, 68.9% had moderate nomophobia, and 18.9% had severe nomophobia [20]. The prevalence rate might differ due to sample size (comparatively large sample size in the present study) and the use of convenience sampling in both studies.

In contrast to the present study's findings, almost twice the prevalence rate of severe nomophobia (i.e., 65%) was reported among university students in Oman (n = 735) [6]. However, the present findings of the study are very similar to a study among Indian university students (n = 209) which reported 35.4% severe nomophobia [7]. The prevalence rate of severe nomophobia (22.1%) was reported to be lower among Saudi Arabian university students (n = 625) [8]. Moreover, a 42.6% severe nomophobia was reported among Turkish college students (n = 537) [37], and a 13% severe nomophobia was reported among a large adult population in Australia (n = 2773) [38]. Therefore, the level of severe nomophobia appears to be relatively high among university students in Bangladesh given all the studies used the NMP-Q. The different rates might be differed due to the sample sizes, different target populations, the use of convenience sampling techniques, and the cultural characteristics of each different country.

Duration of using a smartphone (in daily number of hours) was significantly associated with nomophobia level [7,8]. More specifically, using a smartphone for three or more hours daily was associated with increased severity of nomophobia. According to Bartwal and Nath [13], 21.1% of Indian medical students (n = 451) had severe nomophobia when they used their smartphone for three or more hours daily, whereas the rate was 11.1% among those using their smartphone for less than 3 h daily. Similarly, another study among Saudi Arabian university students (n = 625) reported that severe nomophobia was 30.8% among those using their smartphones for more than 2 h daily which was significantly higher than reported for using their smartphone for less than 1 h daily (7.1%) [8]. However, another study with Indian medical students (n = 451) reported severe nomophobia between those that used their smartphones three or more hours daily (21.1%) compared to those who used their smartphones less than 3 h daily (21.1% vs 11.1%) [13]. These findings are in line with those of the present study suggesting a proportional relationship between nomophobia and number of daily hours spent on smartphones.

Additionally, being in a relationship significantly predicted nomophobia in the present study. This might be due to individuals spending more time on social media and texting [14] to connect with their loved ones using a smartphone [39] which increased the risk of being nomophobic [14]. The present study also found psychoactive substance use to be a predictor of nomophobia. Similarly, a study among young Italian adults (n = 403) found that substance use was a significant predictor of nomophobia. Another study among Indian medical students (n = 246) also reported a significant association between substance use and nomophobia [40]. This might be because students at risk of SA are more likely to use maladaptive strategies such as substance use and smartphone use as a coping strategy to relieve stress [[40], [41], [42]]. However, different findings were reported among Turkish university students (n = 386) where there was no significant association between alcohol/drug use and nomophobia [43].

The present study confirmed the direct effect of FA on SA and nomophobia. A previous study among the Philippines high school students (n = 1447) also demonstrated that nomophobia and SA had a bi-directional relationship [21]. That is, SA can lead to experiencing severe nomophobia and experiencing more severe nomophobia can lead to SA. Additionally, smartphone-related factors such as smartphone checking frequency, daily time spent on a smartphone, and purpose of using it significantly predicted nomophobic behavior among Turkish adolescents [22]. Another study reported that SA was a significant predictor of nomophobia among Turkish nursing students (n = 215) [23]. However, there has only been limited research examining the association between nomophobia and FA. Lin et al. [44] reported a moderate association between nomophobia and the addictive use of social media, whereas in a study of middle and high school students in Morocco (n = 541) reported a significant association between nomophobia and FA [24]. Additionally, negative association between nomophobia and FA was reported among students (n = 348) at a health training institute in Nigeria [45].

Nomophobia was associated with insomnia in the present study. This concurs with the findings of previous studies [[46], [47], [48]]. More specifically, a positively weak correlation was reported between nomophobia and insomnia among the esports players (n = 216) and non-esports players (n = 677) in Saudi Arabia [46]. Additionally, a study among young adults in Bahrain (n = 654) reported a strong relationship between nomophobia and insomnia symptoms [47]. This consistent finding may be due to continuous smartphone use before bedtime leading to less sleep time and poor sleep quality [49]. The present study also found a significant association between nomophobia and depression. These findings concur with previous studies among 1386 Indian high school students (n = 1386) [16] and Peruvian medical students (n = 3139) [50].

### 4.1 Strengths and limitations

The study is the first in Bangladesh to simultaneously report the prevalence, correlates, and predictive factors of nomophobia. In addition to the novelty, the study has a number of strengths: (i) it used standardized and robust psychometric instruments to assess the key variables including nomophobia, Facebook addiction, and mental health factors; (ii) it had comparatively large sample size compared to most previously conducted studies on nomophobia; and (iii) it used mediation model to explain the relationship between smartphone addiction, Facebook addiction, and nomophobia. In addition, the higher prevalence of nomophobia highlights the importance of raising awareness about the negative consequences of nomophobia. It is expected that the present study will help to facilitate further study to be conducted in the country.

Despite these strengths, the present study has some limitations. The study was cross-sectional using a non-probability sampling technique. Therefore, causality between the variables cannot be established and the generalizability of the findings is arguably limited. Future studies should consider longitudinal methods and more rigorous sampling techniques (i.e., probability sampling) to improve the methodological weaknesses of the present study. Moreover, the study was conducted at a single university. Therefore, a multicenter study with a countrywide representative sample size is required to investigate nomophobia among university students as well as other population groups. Furthermore, the number of first-year students was small because they were not on campus during data collection.

## 5 Conclusion

The prevalence of nomophobia is relatively high among Bangladeshi university students and was significantly associated with both SA and FA, as well as with other negative consequences (e.g., insomnia, depression). In addition, the duration of daily time spent on smartphones, psychoactive substance use, and being in a relationship were the predictors of nomophobia. Therefore, reducing daily smartphone use time, and not using psychoactive substances might reduce nomophobia prevalence among university students.

## Author contribution statement

Firoj Al-Mamun: Conceived and designed the experiments; Performed the experiments; Analyzed and interpreted the data; Contributed reagents, materials, analysis tools or data; Wrote the paper.

Mohammed A Mamun; Mark D. Griffiths; Mohammad Muhit; Salauzzaman Prodhan; Md. Muktarul: Contributed reagents, materials, analysis tools or data; Wrote the paper.

Md. Tajuddin Sikder: Conceived and designed the experiments; Contributed reagents, materials, analysis tools or data; Wrote the paper.

## Funding statement

This research did not receive any specific grant from funding agencies in the public, commercial, or not-for-profit sectors.

## Data availability statement

Data will be made available on request.

## Declaration of interest's statement

The authors declare no competing interests.
